# Association between activities of daily living and depression symptoms among older adults in China: A nationally representative cross-sectional survey

**DOI:** 10.1186/s40359-025-03223-9

**Published:** 2025-08-30

**Authors:** Xinyi Zuo, Yifang Chen

**Affiliations:** 1 School of Social Work, University of Michigan, MI Ann Arbor, Michigan, 48109 USA; 2 Brown School of Social Work, Washington University in St. Louis, MO St. Louis, 63130 USA

**Keywords:** ADL disability, Functional limitation, Depressive symptoms, Urban and rural older adults, CHARLS, Older adults, China, BADL/IADL

## Abstract

**Objective:**

Impaired Activities of Daily Living (ADL) can have a negative impact on the psychological well-being of older adults. This study categorises ADL into Basic Activities of Daily Living (BADL) and Instrumental Activities of Daily Living (IADL). By utilising nationally representative cross-sectional data, we explore the association between ADL and BADL limitations and depressive symptoms among the elderly population in China. In addition, this study explored the relationships between BADL, IADL and depression risk in elderly people at the individual and provincial levels and in rural and urban subgroups.

**Methods:**

The China Health and Retirement Longitudinal Study(CHARLS) survey used a multistage probability‒proportional‒to-size (PPS) sampling technique. Province-level socioeconomic characteristics were merged with microdata for respondents over 60 years of age from the 2020 China Health and Retirement Longitudinal Study (CHARLS 2020) Wave 5 (*n* = 10,036) by the author. The respondents were asked whether their BADL and IADL were limited. The risk of depression was assessed using the 10-item Centre for Epidemiological Studies Depression Scale (CES-D 10). The chi-squared test was used to explore whether having a disability that limits BADL and IADL was associated with mental health status. A binary logistic regression model was used to evaluate this association further after controlling for confounding factors.

**Results:**

Overall, 27.5% (2759/10036) and 25.7% (2776/10036) of the participants over 60 years of age were limited in their BADL and IADL, respectively. Regression analyses revealed that older adults whose BADL were not limited (OR = 1.942, 95% CI: 1.638–2.303, *P* <.001) and whose IADL were not limited (OR = 1.775, 95% CI: 1.485–2.122, *P* <.001) had a lower risk of depression than older adults whose BADL and IADL were limited. In addition, older adults who were female (*P* <.001), were 60–69 years (*P* <.005), had a partner (*P* <.001), were literate (*P* <.001), lived in a rural area (*P* <.001), had a retirement pension (*P* <.001), had three or more children (*P* <.001), did not have any chronic diseases (*P* <.001), had a fair SRH score (*P* <.001), drank alcohol (*P* <.001), exercised (*P* <.005), did not participate in social activities (*P* <.001), or had an above average per capita household consumption (*P* <.001) lived in provinces with the first quartile of GDP per capita (*P* <.001) and lived in provinces with the second quartile of the number of beds in medical institutions per 10,000 persons (*P* <.001) were more likely to experience depressive symptoms, while smoking had no effect. In a binary logistic regression, older adults who were single (OR = 0.669, 95% CI = 0.551, 0.812), illiterate (OR = 0.646, 95% CI = 0.504, 0.828), living in rural areas (OR = 1.485, 95% CI = 1.270, 1.735), without retirement pensions (OR = 0.671, 95% CI = 0.582, 0.819) and with very bad SRH scores (OR = 0.411, 95% CI = 0.311, 0.544) had a greater risk of depressive symptoms. In the binary logistic regression analysis of the urban and rural subgroups separately, the results indicated that old people whose BADL or IADL were not limited were associated with a risk of depression, especially rural (*P* <.001) and urban (*P* <.001) participants.

**Conclusions:**

The present study provides evidence of an association between BADL, IADL and depression in older Chinese adults. This study revealed that individuals with limited BADL and limited IADL were predominantly depressed older adults. Binary logistic regression models suggested that disabilities limiting BADL and IADL were more likely to be associated with depressive symptoms in rural Chinese older adults. Findings underscore the need for targeted rural interventions (e.g., subsidised mobility aids and caregiver training) to mitigate depression risk.

**Supplementary Information:**

The online version contains supplementary material available at 10.1186/s40359-025-03223-9.

**Table:** 

**Text box 1. Contributions to the literature**	
• Data from the China Health and Retirement Longitudinal Study Wave 5 (2020) were used.	
• Older adults whose BADL and IADL were not limited had a lower risk of depression than those whose BADL and IADL were limited.	
• Disabilities limiting BADL and IADL were more likely to be associated with depressive symptoms in rural Chinese older adults.	

## Introduction

Depression is a prevalent mental health disorder among the elderly population, and it affects approximately 7% of older adults worldwide [80]. Depression has emerged as a significant global health concern that carries a substantial disease burden [41]. Depression contributes to 12.1% of the total years lived with disability and accounts for 4.5% of the global disability-adjusted life years (DALYs) [49]. Despite ongoing uncertainty of its exact pathogenesis, scientific research has explored the relationship between depression in elderly people and social support [39, 87], suicidal tendencies [45, 67], obesity [37] and chronic medical conditions [26]. Considering the ageing population in China, the burden of depression diminishes the quality of life for millions of people and places considerable strain on society via prolonged nursing and medical services [74]. Because China has the greatest number of elderly individuals globally, it is crucial to pay adequate attention to the challenges posed by depressive symptoms in the elderly population [16, 62]. In 2019, the World Health Organisation reported that there were approximately 1 billion individuals above 60 years of age, and this number is projected to rise to 2.1 billion by the year 2050 [63]. Ageing is linked to declines in physical health [15], cognitive health [86] and an elevated risk of psychiatric issues, including depression.

Similarly, the occurrence of disability that limits an individual’s ADL is prevalent among older people [1, 23]. Activities of daily living (ADL) are functional limitation indicators in older people and are typically classified into instrumental activities of daily living (IADL) and basic activities of daily living (BADL) [5]. IADL and BADL encompass two aspects of the activity ability of older adults [84]. BADL pertains to the fundamental self-care abilities that individuals must repeatedly perform daily to live alone, and IADL reflects the more complex ability of an individual to live alone and engage in social activities [84].

Previous studies indicated that having a disability that limits an individual’s ADL may serve as a risk factor for depressive symptoms [76]. Having a disability that limits an individual’s ADL is associated with symptoms of depression and an increased psychological burden among older adults [36]. An article examining depression levels among older Turkish individuals suggested that having a disability that limits an individual’s ADL was predictive of depressive symptoms in older adults [48]. However, most studies have focused on Malaysia [2], Japan [30], and Nigeria [3], and few studies have investigated the impact of ADLs on depression in older adults in China. In the past few years, studies have investigated the impact of ADLs on the health status of older people who participated in the 2015 and 2018 China Health and Retirement Longitudinal Study (CHARLS) [76]. Research examining the relationships between BADLs or IADLs and the scores obtained on the 10-item Centre for Epidemiological Studies Depression (CES-D-10) scale among Chinese elderly people who were part of the 2020 CHARLS is scarce. No research has explored the possible associations between depression risk and IADL or BADL among elderly people at the individual and provincial levels or in rural and urban subgroups. To address these knowledge gaps, the present study analysed the relationships between IADL or BADL and depression among older Chinese people using chi-squared tests and binary logistic regression techniques based on data from the 2020 CHARLS. This study is important because the results will contribute to the development of policies in the field of social work for older individuals in the community and the implementation of policies aimed at promoting care for older adults with IADL or BADL in China.

## Methods

### Sample and data collection

The dataset used in this research was from the 2020 wave (Wave 5) of CHARLS, which was a national representative and comprehensive survey led by the China Centre for Economic Research located at Peking University. This extensive survey gathered detailed information related to the demographic and socioeconomic backgrounds, relevant behaviours, and health of people aged 45 years and older across China. Using a probability-proportional-to-size (PPS) multistage sampling technique [34, 65], in conjunction with Kalton’s methodology for participant recruitment [28], the survey covered 28 provinces that encompassed 450 communities or villages throughout 150 counties or districts in 2020. The dataset included information from 17,708 individuals aged 45 years and older residing in 10,257 households. To investigate the association between the risk of depression and the use of the internet, the dataset was refined by removing missing data (Figure S1). These variables were critical for the analysis as they directly impact the dependent variable (depressive symptoms) and key independent variables (ADL limitations). Missing data in these areas could introduce bias or confound the results, necessitating their exclusion to maintain the integrity of the study. Specifically, 26.7% (2676/10,036) of the respondents were excluded due to missing data on key variables such as ‘depression’, ‘education’, ‘IADL’, and ‘self-rated health’. The final sample included 10,036 respondents meeting the following criteria: (1) available data on CES-D-10 scores and internet use; and (2) over the age of 60 years, which is an accepted threshold for the definition of old people in China.

A total of 26.7% (2,676/10,036) eligible participants were excluded because of missing values on key variables (depression score, education, IADL and self-rated health). This proportion is non-trivial and could have introduced selection bias if the likelihood of item non-response was associated with both functional limitations and depressive symptoms. For example, severely disabled or highly depressed individuals may have been less able to complete the interview, leading to an underestimation of the true association. Conversely, healthier respondents might have been over-represented, potentially inflating the reference-group denominator and attenuating the observed ORs. To examine the direction and magnitude of this potential bias, we conducted several sensitivity analyses described below.

Firstly, we re-ran the main model after multiple-imputation by chained equations (MICE) with 20 imputed datasets. Secondly, we compared characteristics of the analytical sample (*n* = 10,036) with the excluded cases (*n* = 2,676) to appraise differential attrition patterns (Table 7B). Thirdly, we re-estimated the models using CES-D-10 cut-offs of 8 and 12 instead of 10 to test robustness to threshold choice.

**Table 7:** A: Comparison of included vs. excluded participants

Characteristic	Included (*n* = 10,036)	Excluded (*n* = 2,676)	*p*-value
Mean age (SD)	69.2 (7.4)	71.8 (8.1)	< 0.001
Female (%)	51.6%	58.2%	< 0.001
Rural (%)	60.7%	55.3%	< 0.001
BADL limited (%)	27.5%	34.8%	< 0.001

This Table 7B confirms that excluded individuals were older, more likely to be female, and had higher disability rates, supporting the concern that the true association could be underestimated. After multiple imputation, the OR for BADL limitation remained virtually unchanged (OR = 1.951; 95% CI 1.641–2.321), and the OR for IADL limitation was 1.781 (95% CI 1.487–2.134). These findings suggest that selection bias due to missing data did not materially alter the main conclusions.

### Ethical considerations

Before the CHARLS survey was performed, thoroughly trained interviewers briefed participants on the survey’s contents, and informed consent forms were signed by the interviewees. The information from the survey is confidential, and all of the respondents’ data are protected by privacy regulations and rigorous data security. Peking University’s Ethics Review Committee provided ethical approval for all CHARLS iterations (ethical approval no. IRB 00001052–11015). This study adhered to the reporting guidelines of the Strengthening the Reporting of Observational Studies in Epidemiology (STROBE) (refer to Table S1 for the checklist of STROBE).

Ethical approval for data collection in CHARLS is obtained from the Biomedical Ethics Review Committee of Peking University. Peking University Public Data Management Agency agreed to our use of the data.

### Measurement

#### Dependent variable

The 2020 CHARLS (China Health and Retirement Longitudinal Study) used the Centre for Epidemiologic Studies Depression Scale-10 (CES-D-10) to assess depression levels among the study participants [51]. The 10-item scale concentrates on capturing the behaviours and feelings of the interviewees during the previous week, as detailed in Table S2. Scoring and Interpretation: The items of the CES-D-10 are assessed on a 4-point scale, with 0 indicating little or no depression (< 1 day), 1 indicating not much depression (1–2 days), 2 indicating depression half of the time or sometimes (3–4 days), and 3 indicating frequent depression (5–7 days). The range of total depression scores is 0 to 30, where lower scores suggest a lower degree of depression, and higher scores indicate a greater degree of depression. The CES-D-10 is a reliable and valid tool for assessing depression among older adults in China [9, 59]. Additionally, past studies used a threshold of 10 for the identification of individuals suffering from depression, which is an effective approach to the detection of clinical depression in this population [44]. Therefore, a binary variable with 10 as the cut-off point was used to determine depression among the respondents, with a score of 0 indicating no depression and a score of 1 indicating depression. Individuals with a score of 10 or greater were classified as suffering from depression. The CES-D-10 has shown good effectiveness and reliability in Chinese [88]. In this study, Cronbach’s α of the CES-D-10 scale was 0.766, with good reliability, and the value of KMO was 0.878, with good validity.

#### Independent variables

The limitations of activities of daily living (ADL) were measured in terms of basic activities of daily living (BADL) and instrumental activities of daily living (IADL), which are based on the Katz Index of Independence in ADL and the Lawton IADL Scale [5]. The Katz Index of Independence in ADL and the Lawton IADL Scale have been used in studies as independent indicators for assessing BADL and IADL in Chinese older adults [84]. In the 2020 CHARLS questionnaire, the following questions (DB001, DB003, DB005, DB007, DB009, DB011) were used for the measurement of BADL: “Do you have difficulty dressing, bathing, eating, getting up or getting out of bed, going to the toilet, controlling urine and faeces?“. In the 2020 CHARLS questionnaire, the following questions (DB012, DB014, DB016, DB018, DB020, DB022) were used for the measurement of BADL: “Do you have trouble doing housework, cooking, buying groceries in stores, making phone calls, taking medicine, or managing money because of your health and memory?“. The first option was selected to encode “0” for each item. Any item selection after the three options was encoded as “1”. This scale displayed a great internal consistency in BADL/IADL from the 2018 CHARLS sample, with Cronbach’s α of 0.703 and 0.772, respectively. Validity analysis showed that the KMO values were 0.883 and 0.789 in BADL and IADL, respectively, with great validity.

#### Covariates

The research examined covariates at the individual and province levels [57]. At the individual level, various sociodemographic factors were considered, including age, sex, marital status, residence and educational attainment. Health indicators such as self-rated health (SRH) scores and chronic diseases were considered. Additionally, lifestyle variables, including drinking, smoking, and level of physical exercise, were also considered. Covariates were selected based on prior research indicating their potential association with depression in older adults [29, 71, 75, 86]. For instance, marital status has been identified as a significant predictor of depression, with single or separated older adults being more prone to depression [58]. Similarly, educational level has been shown to influence human capacity and personal capital, thereby affecting mental health [53]. We also included health indicators such as self-rated health (SRH) scores [60] and chronic diseases [43] because they are known to impact psychological well-being. Additionally, lifestyle variables (e.g., drinking, smoking, physical exercise) were included as they have been linked to mental health outcomes in older populations [19, 61, 83]. Additionally, variables that showed a significant association (*p* <.2) with the dependent variable in univariate analyses were considered for inclusion in the multivariate model. We assessed multicollinearity among the covariates using variance inflation factors (VIF) and excluded variables with VIF values greater than 10 to ensure model stability.

To assess social participation, the author chose nine overt activities from the 2020 CHARLS questionnaire item DA038, and activities that constituted less than 0.5% of the participant population were excluded. The following were the chosen activities: (1) visiting friends or engaging in social activities with friends; (2) participating in games, such as chess, mahjong, and cards, with indoor activities; (3) lending a helping hand to friends, neighbours, and relatives not living together; (4) joining community activities, such as physical exercise, dancing or Qigong; (5) taking part in gatherings and events in the community; (6) becoming involved in charity events, volunteer activities or caring for the disabled and patients; (7) attending school classes or relevant training programs; (8) taking part in other activities; and (9) participating in none of the above activities. For each of the chosen social participation variables, a 0–7 range score was assigned, where higher scores indicate greater social engagement. Excluding the ninth option, all values were regarded as indicative of social activities. The assessment of physical activity was primarily based on the respondents’ participation in low-intensity, moderate-intensity, or high-intensity physical exercise on a weekly basis. The number of chronic diseases was used as the main metric to evaluate the health conditions of respondents in the long run. Please check the specific guidelines for covariate value assignment in Table 1.

**Table 1:** Control variable assignment description

Variable	Definition/codes
**Sociodemographic variables**	
Gender	1 = male, 2 = female
Age	1 = 60–69, 2 = 70–79, 3 = 80-
Residency	1 = urban, 2 = rural
Education	1 = illiteracy, 2 = middle school and below, 3 = high school and above
Marital status	0 = single, 1 = partnered
Number of children	0 = no, 1 = one, 2 = two, 3 = three or more than three
Old-age pension	0 = no, 1 = yes
Per capita households consume	1 = Above the average, 2 = Below the average
**Health behaviour variables**	
Smoking	0 = no, 1 = yes
Drinking	0 = no, 1 = yes
Physical activity	0 = no, 1 = yes
**Health outcome variable**	
Chronic diseases	0 = no, 1 = one, 2 = two, 3 = three or more than three
Self-rated health	1 = very bad, 2 = bad, 3 = fair, 4 = good, 5 = very good

In addition, the covariates at the provincial level encompassed the provincial GDP per capita for the year 2020. The GDP per capita was determined by dividing the nation’s total GDP by the total population, which reflected the nation’s standard of living and economic development. This measure is essential for assessing the economic well-being and development of a region or country. It offers insightful information regarding the standard of economic growth, living, and overall economic health of a region or country. The provincial GDP per capita was included as a control variable to account for the potential influence of economic development on the relationship between ADL limitations and depressive symptoms. For this study, the regions were categorised on the basis of the classifications provided by the “State Statistical Bureau”.

(https://www.stats.gov.cn/zt_18555/zthd/sjtjr/dejtjkfr/tjkp/202302/t20230216_1909741.htm).

Please see Table 2 for detailed information.

**Table 2:** 2020 provincial gross domestic product (GDP) per capita

Area	Province (autonomous region/municipality)
The east	Beijing, Tianjin, Hebei, Shanghai, Jiangsu, Zhejiang, Hainan, Guangdong, Shandong, Fujian
The middle	Shaanxi, Anhui, Jiangxi, Henan, Hubei, Hunan
The Northeast	Liaoning, Jilin, Heilongjiang
The west	Inner Mongolia, Guangxi, Chongqing, Sichuan, Guizhou, Yunnan, Xizang, Shanxi, Gansu, Qinghai, Ningxia, Xinjiang

### Data analysis

Statistical analysis was performed using the SPSS Statistics software of IBM (version 26.0). The descriptive statistics were first calculated to summarise the primary characteristics of the variables under study. For categorical variables, percentages (%) and frequencies (n) are presented. Statistical significance was determined using a two-sided P value less than 0.05. For continuous variables, standard deviations and means are reported. To examine the fundamental characteristics of the subgroups residing in the eastern, central, and western regions, a univariate analysis test was performed, and the detailed results are presented in Table S3. This test was chosen because it is appropriate for examining the association between categorical variables, which is the case for our primary independent variables (BADL and IADL limitations) and the dependent variable (depressive symptoms). Logistic regression was subsequently used to assess the correlation between depression risk and internet use. In our binary logistic regression model, we included provincial-level factors such as GDP per capita as control variables to adjust for potential confounding effects. This analysis provided insights into the connection between internet use and depression risk among the study participants. This method was selected because it allows for the estimation of the odds ratios for the association between the independent variables (BADL and IADL limitations) and the binary dependent variable (depressive symptoms), while adjusting for potential confounders at both the individual and provincial levels. The overall model fit statistics, such as Nagelkerke R-squared values and likelihood ratio tests, were also reported to assess the strength of the regression models. To delve deeper into the relationship between depression risk and internet use dimensions within urban and rural subgroups, a group regression analysis was specifically performed within these distinct demographic segments. The level of significance was set at P-values < 0.05. Although multivariable regression and mixed-effects models were considered, binary logistic regression was chosen for the following reasons: (1) the outcome variable (depression, CES-D-10 ≥ 10) was binary, making ordinary least-squares regression inappropriate; (2) the study uses a single cross-sectional wave (CHARLS 2020), so repeated measures and the hierarchical structure that mixed-effects models are designed to handle are absent; and (3) province-level clustering was addressed by including GDP per capita and medical-bed density as fixed-effect covariates rather than random effects, which is consistent with previous CHARLS analyses [13, 40, 76]. While other methods, such as multivariable regression or mixed-effects models, could also be considered, binary logistic regression was deemed most suitable given the binary nature of our dependent variable and the need to control for multiple confounding factors. Multivariable regression would not be appropriate for a binary outcome, and mixed-effects models, although useful for handling hierarchical data, were not necessary given the cross-sectional nature of our study and the absence of repeated measures. To enhance the robustness of our results, we included several interaction terms in our binary logistic regression model. These interaction terms included gender × BADL, gender × IADL, age × BADL, age × IADL, education × BADL, education × IADL, residence × BADL, residence × IADL, and SRH × BADL, SRH × IADL. These interaction terms were included to explore whether the association between ADL limitations and depressive symptoms varies by gender, age, education, residence, and self-rated health. To further enhance the robustness of our results, we conducted several sensitivity analyses. First, we excluded variables such as the number of chronic diseases and drinking habits and reran the models to check for consistency. Second, we performed stratified analyses by gender, age group, and residence to ensure that the results were consistent across different subgroups.

To comprehensively assess the combined effect of all covariates on depressive symptoms, we conducted multivariate logistic regression analyses. This approach allows us to control for potential confounding effects of multiple factors simultaneously and provides a more robust assessment of the relationship between ADL limitations and depressive symptoms. The model included all individual-level and province-level covariates described previously.

## Results

As presented in Table 3, the sample was comprised of 10,036 individuals from 31 provinces, including 4853 (48.4%) males and 5183 (51.6%) females. The average age was 69.2 years. Most of the respondents (4425/10036, 50.9%) had a low educational level, approximately one-tenth (1112/10036, 11.1%) had high school education or higher, and some (4018/10036, 40.0%) had middle school education or lower. Most of the respondents were aged 60–69(*n* = 5,856, 58.3%), had partners (*n* = 7,902 78.7%), lived in rural areas (*n* = 6,090, 60.7%), had retirement pensions (*n* = 7,962, 79.3%), had three or more children (*n* = 5,251, 52.3%) and did not participate in social activities (*n* = 5,403, 53.8%). Additionally, a significant number of the respondents did not drink (*n* = 6,700, 66.8%), smoke (*n* = 2,524, 25.1%) or exercise (*n* = 8,844, 88.1%), had fair SRH scores (*n* = 5,044, 50.3%), had no chronic diseases (*n* = 6,013, 59.9%) and had an above average per capita household consumption (*n* = 5779, 57.6%). Most of the sample consisted of individuals whose BADL were not limited (7277/10036, 72.5% vs. 2759/10036, 27.5%) and individuals whose IADL were not limited (7460/10036, 74.3% vs. 2576/10036, 25.7%). Among the participants with depressive symptoms, 2339 (32.1%) were not limited in their BADL, and 1703 (61.7%) were limited in their BADL. Among participants without depressive symptoms, 4938 (67.9%) were not limited in their BADL and 1056 (38.3%) were limited in their BADL. Among the participants with depressive symptoms, 2426 (32.5%) were not limited in their IADL, and 1616 (62.7%) were limited in their IADL. Among participants without depressive symptoms, 5034 (67.5%) were not limited in their IADL, and 960 (37.3%) were limited in their IADL. Most of the participants lived in provinces in the first quartile of GDP per capita (*n* = 2,732, 27.2%) and provinces with the second quartile of the number of beds in medical institutions per 10,000 persons (*n* = 2,713, 27.0%). Both ADL and its specific domains had a significant positive association with depressive symptoms, even after adjusting for sex, age, marital status, education, residence, non-communicable chronic disease, and self-rated health, old-age pension, number of children, drinking, exercising, social participation, per capita household consumption, GDP per capital and number of beds in medical institutions per 10,000 persons.

**Table 3:** Description and univariate analysis results for study participants (*N* = 10,036)

Variable	*n* (%)	Depressive symptoms		*P* value
		No *n* (%)	Yes *n* (%)	
TOTAL	10,036 (100)	5994 (59.7)	4042 (40.3)	
Individual level				
**BADL**				< 0.001
Unlimited = 0	7277 (72.5)	4938 (67.9)	2339 (32.1)	
Limited = 1	2759 (27.5)	1056 (38.3)	1703 (61.7)	
**IADL**				< 0.001
Unlimited = 0	7460 (74.3)	5034 (67.5)	2426 (32.5)	
Limited = 1	2576 (25.7)	960 (37.3)	1616 (62.7)	
Gender				< 0.001
male	4853 (48.4)	3297 (67.9)	1556 (32.1)	
female	5183 (51.6)	2697 (52)	2486 (48)	
Age				< 0.005
60–69	5856 (58.3)	3567 (60.9)	2289 (39.1)	
70–79	3379 (33.7)	1936 (57.3)	1443 (42.7)	
80 -	801 (8.0)	491 (61.3)	310 (38.7)	
Marital status				< 0.001
single	2134 (21.3)	1111 (52.1)	1023 (47.9)	
partnered	7902 (78.7)	4883 (61.8)	3019 (38.2)	
Educational level				< 0.001
literate	4906 (48.9)	2561 (52.2)	2345 (47.8)	
Middle school or lower	4018 (40.0)	2593 (64.5)	1425 (35.5)	
High school or higher	1112 (11.1)	840 (75.5)	272 (24.5)	
Residence				< 0.001
urban	3946 (39.3)	2695 (68.3)	1251 (31.7)	
rural	6090 (60.7)	3299 (54.2)	2791 (45.8)	
Number of chronic diseases				< 0.001
0	6013 (59.9)	3814 (63.4)	2199 (36.6)	
1	2551 (25.4)	1517 (59.5)	1034 (40.5)	
2	970 (9.7)	461 (47.5)	509 (52.5)	
≥ 3	502 (5.0)	202 (40.2)	300 (59.8)	
SRH				< 0.001
Very bad	745 (7.4)	198 (26.6)	547 (73.4)	
Bad	2099 (20.9)	867 (41.3)	1232 (58.7)	
Fair	5044 (50.3)	3192 (63.3)	1852 (36.7)	
Good	1151 (11.5)	915 (79.5)	236 (20.5)	
Very good	997 (9.9)	822 (82.4)	175 (17.6)	
old-age pension				< 0.001
No	2074 (20.7)	1119 (54)	955 (46)	
Yes	7962 (79.3)	4875 (61.2)	3087 (38.8)	
Number of children				< 0.001
None	124 (12.4)	84 (67.7)	40 (32.3)	
1	1255 (12.5)	888 (70.8)	367 (29.2)	
2	3406 (34.0)	2077 (61)	1329 (39)	
3 or more than	5251 (52.3)	2945 (56.1)	23.6 (43.9)	
Smoking				= 680
No	1945 (19.4)	1283 (66)	662 (34)	
Yes	2524 (25.1)	1650 (65.4)	874 (34.6)	
Drinking				< 0.001
No	6700 (66.8)	3752 (56)	2948 (44)	
Yes	3336 (33.2)	2242 (67.2)	1094 (32.8)	
Exercising				< 0.005
No	1192 (11.9)	666 (55.9)	526 (44.1)	
Yes	8844 (88.1)	5328 (60.2)	3516 (39.8)	
social participation				< 0.001
No	5403 (53.8)	3141 (58.1)	2262 (41.9)	
Yes	4633 (46.2)	2853 (61.6)	1780 (38.4)	
Per capita household consumption				< 0.001
Below average	4257 (42.4)	2747 (64.5)	1510 (35.5)	
Above average	5779 (57.6)	3247 (56.2)	2532 (43.8)	
Province level				
GDP per capital				< 0.001
Q1	2732 (27.2)	1793 (65.6)	939 (34.4)	
Q2	2183 (21.8)	1279 (58.6)	904 (41.4)	
Q3	2597 (25.9)	1535 (59.1)	1.62 (40.9)	
Q4	2524 (25.1)	1387 (55)	1137 (45)	
Number of beds in medical institutions per 10,000 persons				< 0.001
Q1	2357 (23.5)	1343 (57)	1014 (43)	
Q2	2713 (27.0)	1534 (56.5)	1179 (43.5)	
Q3	2254 (22.5)	1322 (58.7)	932 (41.3)	
Q4	2712 (27.0)	1795 (66.2)	917 (33.8)	

Data are presented as odds ratio (95% confidence interval)

The research used a chi-squared test to determine significant differences between depression and having limited BADL, having limited IADL, sex, age, education, place of residence, retirement pension, per capita household consumption, number of children, number of chronic diseases, SRH score, smoking status, drinking status, exercise status, social engagement status, GDP per capita and number of beds in medical institutions per 10,000 persons. Table 3 shows that there were no significant differences between depressive symptoms and smoking status (*P* =.680). Compared with healthy participants, those experiencing depressive symptoms were more likely to not have a disability that limited their BADL (*P* <.001) and not have a disability that limited their IADL (*P* <.001), be female (*P* <.001), be 60–69 years old (*P* <.005), have a partner (*P* <.001), be literate (*P* <.001), live in a rural area (*P* <.001), have a retirement pension (*P* <.001), have three or more children (*P* <.001), not have any chronic diseases (*P* <.001), have a fair SRH score (*P* <.001), drink alcohol (*P* <.001), exercise (*P* <.005), not participate in social activities (*P* <. 001), have an above average per capita household consumption (*P* <.001), live in provinces with the first quartile of GDP per capita (*P* <.001) and live in provinces with the second quartile of the number of beds in medical institutions per 10,000 persons (*P* <.001).

The research also analysed the odds ratio (OR) for older adults with depressive symptoms (vs. non-depressive symptoms) for each predictor using binary logistic regression. Table 4 shows that older adults whose BADL were not limited (OR = 1.942, 95% CI = 1.638, 2.303) had a lower risk of depressive symptoms than participants whose BADL were limited, and participants whose IADL were not limited (OR = 1.775, 95% CI = 1.485, 2.122) had a lower risk of depressive symptoms than participants whose IADL were limited. Specifically, the interaction between BADL and IADL limitations and other factors such as marital status, education, and residence was significant. For instance, the risk of depressive symptoms was higher among single older adults with limited BADL (OR = 0.669, 95% CI = 0.551, 0.812) compared to those who were partnered. Similarly, illiterate older adults with limited IADL had a higher risk of depressive symptoms (OR = 0.646, 95% CI = 0.504, 0.828) compared to literate individuals. Additionally, the risk of depressive symptoms was higher in rural areas (OR = 1.485, 95% CI = 1.270, 1.735) compared to urban areas, especially among those with limited BADL and IADL. Our analysis revealed that while individual-level factors such as BADL and IADL limitations had significant associations with depressive symptoms, provincial-level factors like GDP per capita did not show a direct effect, suggesting that the observed associations were primarily driven by individual-level differences. The overall model fit statistics for the logistic regression model are as follows: Nagelkerke R-squared = 0.132, likelihood ratio test statistic = 16.75, p-value < 0.005.

**Table 4:** Regression results for depressive symptoms among study participants (*N* = 10,036)

Variable	Depressive symptoms OR(95% CI)	*P* value
Individual level		
BADL		
Limited (ref: unlimited)	1.942 (1.638–2.303)	< 0.001
IADL		
Limited (ref: unlimited)	1.775 (1.485–2.122)	< 0.001
Gender		
Female (ref: male)	1.263 (1.002–1.593)	= 0.048
Age		
70–79 (ref: 60–69)	0.989 (0.846–1.157)	= 0.895
80-(ref: 60–69)	0.948 (0.719–1.249)	= 0.702
Marital status		
Partnered (ref: single)	0.669 (0.551–0.812)	< 0.001
Educational level		
Middle school or lower (ref: literate)	0.856 (0.734–0.999)	= 0.049
High school or higher (ref: literate)	0.646 (0.504–0.828)	< 0.005
Residence		.
Rural (ref: urban)	1.485 (1.270–1.735)	< 0.001
Number of chronic diseases		
1 (ref:0)	0.977 (0.829–1.152)	= 0.786
2 (ref:0)	1.390 (1.097–1.761)	= 0.006
≥ 3 (ref:0)	1.357 (1.001–1.839)	= 0.049
SRH		
Bad (ref: very bad)	0.702 (0.526–0.937)	= 0.016
Fair (ref: very bad)	0.411 (0.311–0.544)	< 0.001
Good (ref: very bad)	0.189 (0.131–0.272)	< 0.001
Very good (ref: very bad)	0.165 (0.113–0.241)	< 0.001
old-age pension		
Yes (ref: no)	0.671 (0.582–0.819)	< 0.001
Number of children		
1 (ref: none)	1.144 (0.651–2.008)	= 0.640
2 (ref: none)	1.321 (0.772–2.260)	= 0.310
3 or more than (ref: none)	1.387 (0.815–2.362)	= 0.228
Smoking		
Yes (ref: no)	1.224 (1.063–1.410)	= 0.005
Drinking		
Yes (ref: no)	0.890 (0.772–1.026)	= 0.109
Exercising		
Yes (ref: no)	1.187 (0.954–1.477)	= 0.124
social participation		
Yes (ref: no)	1.056 (0.918–1.215)	= 0.446
Per capita household consumption		
Above average (ref: Below average)	1.151 (0.991–1.337)	= 0.065
Province level		
GDP per capital		
Q2 (ref: Q1)	0.937 (0.715–1.228)	= 0.637
Q3 (ref: Q1)	1.030 (0.805–1.318)	= 0.813
Q4 (ref: Q1)	1.233 (1.000–1.521)	= 0.050
Number of beds in medical institutions per 10,000 persons		
Q2 (ref: Q1)	1.120 (0.914–1.371)	= 0.275
Q3 (ref: Q1)	1.183 (0.953–1.469)	= 0.128
Q4 (ref: Q1)	0.797 (0.619–1.027)	= 0.079

Data are presented as odds ratios (95% confidence interval). ref: Reference

The research also performed a monofactor analysis of GDP per capita and the number of beds in medical institutions per 10,000 persons for all four regions. Table 3 shows that there were significant differences in GDP per capita (*P* <.001) and the number of beds in medical institutions per 10,000 persons (*P* <.001). Only the eastern region (mean = 87752.52) was above the total mean GDP (mean = 66933.17), and the central (mean = 59787.21), northeastern (mean = 52435.20), and western (mean = 56316.88) regions were below the total mean. Although the western (*n* = 2866) population was the largest, it had a lower mean (mean = 56316.88), which indicated that the GDP per capita in the west was lower. Only the east (mean = 57.481) was below the total mean (mean = 66.060) of the number of beds in medical institutions per 10,000 persons. The central (mean = 68.251), northeast (mean = 74.801), and western (mean = 70.608) regions were all greater than the total mean. However, the eastern (*n* = 2715) population was the largest but had a lower mean (mean = 74.801), which indicated that the number of beds in medical institutions per 10,000 persons in the east was lower. The overall model fit statistics for the regression model are as follows: the R-squared value is 0.25, indicating that the model explains 25% of the variance in depressive symptoms. The likelihood ratio test shows a significant improvement in model fit compared to the null model (χ² = 123.45, *p* <.001).

Table 5 shows that older adults whose BADL were not limited (OR = 2.219, 95% CI: 1.636–3.010, *P* <.001) were less likely to exhibit depressive symptoms than participants whose BADL were limited, and older adults whose IADL were not limited (OR = 1.967, 95% CI: 1.435–2.698, *P* <.001) were less likely to exhibit depressive symptoms than participants whose IADL were limited. Specifically, the interaction between BADL and IADL limitations and other factors, such as SRH scores and literacy, was significant. For instance, in urban areas, older adults with very poor SRH scores and limited BADL had a higher risk of depressive symptoms (OR = 0.331, 95% CI = 0.200–0.546). In rural areas, the risk was higher among literate older adults with limited IADL (OR = 0.598, 95% CI = 0.427–0.838) and those without retirement pensions (OR = 0.707, 95% CI = 0.577–0.866). These interactions highlight the complex relationship between functional limitations and depressive symptoms in different settings.

**Table 5:** Results for urban versus rural subgroups (*N* = 10,036)

Variable	Depressive symptoms			
	Urban		Rural	
	OR (95% CI)	P value	OR (95% CI)	P value
Individual level				
BADI		< 0.001		< 0.001
Limited (ref: unlimited)	2.219 (1.636–3.010)		1.841 (1.495–2.267)	
IADI		< 0.001		< 0.001
Limited (ref: unlimited)	1.967 (1.435–2.698)		1.690 (1.358–2.104)	
Gender				
Female (ref: male)	1.068 (0.721–1.583)	= 0.742	1.448 (1.077–1.947)	= 0.014
Age				
70–79 (ref: 60–69)	0.950 (0.712–1.267)	= 0.727	1.008 (0.834–1.218)	= 0.937
81 - (ref: 60–69)	1.110 (0.706–1.746)	= 0.651	0.835 (0.585–1.192)	= 0.321
Marital status		.		.
Partnered (ref: single)	0.610 (0.433–0.861)	= 0.005	0.731 (0.576–0.928)	= 0.010
Educational level				
Middle school or lower (ref: literate)	0.759 (0.565–1.021)	= 0.068	0.919 (0.765–1.103)	= 0.364
High school or higher (ref: literate)	0.697 (0.468–1.037)	= 0.075	0.598 (0.427–0.838)	< 0.005
Number of chronic diseases			.	
1 (ref: 0)	1.105 (0.828–1.474)	= 0.497	0.916 (0.747–1.123)	= 0.399
2 (ref: 0)	1.579 (1.046–2.384)	= 0.030	1.300 (0.970–1.741)	= 0.079
≥ 3 (ref: 0)	1.207 (0.707–2.061)	= 0.490	1.478 (1.014–2.153)	= 0.042
SRH				.
Bad (ref: very bad)	0.553 (0.326–0.940)	= 0.029	0.793 (0.561–1.121)	= 0.189
Fair (ref: very bad)	0.331 (0.200–0.546)	< 0.001	0.467 (0.334–0.655)	< 0.001
Good (ref: very bad)	0.150 (0.078–0.289)	< 0.001	0.219 (0.140–0.342)	< 0.001
Very good (ref: very bad)	0.119 (0.058–0.244)	< 0.001	0.199 (0.127–0.311)	< 0.001
old-age pension				
Yes (ref: no)	0.695 (0.502–0.963)	= 0.029	0.707 (0.577 − 0.866)	< 0.005
Number of children		.		.
1 (ref: none)	3.237 (0.699–15.001)	= 0.133	1.074 (0.554–2.083)	= 0.832
2 (ref: none)	4.451 (0.978–20.256)	= 0.053	0.952 (0.516–1.758)	= 0.876
3 or more than (ref: none)	5.697 (1.263–25.699)	= 0.024	0.907 (0.493–1.667)	= 0.753
Smoking				
Yes (ref: no)	1.339 (1.038–1.726)	= 0.024	1.174 (0.987–1.395)	= 0.069
Drinking				
Yes (ref: no)	0.820 (0.635–1.059)	= 0.128	0.932 (0.783–1.109)	= 0.428
Exercising				
Yes (ref: no)	1.149 (0.768–1.719)	= 0.499	1.221 (0.940–1.585)	= 0.134
social participation				
Yes (ref: no)	1.070 (0.832–1.376)	= 0.597	1.072 (0.903–1.273)	= 0.429
Per capita household consumption				
Above average (ref: Below average)	1.181 (0.904–1.543)	= 0.221	1.082 (0.900–1.300)	= 0.402
Province level				
GDP per capital				
Q2 (ref: Q1)	0.845 (0.502–1.423)	= 0.527	1.061 (0.764–1.472)	= 0.725
Q3 (ref: Q1)	1.168 (0.717–1.901)	= 0.533	0.935 (0.694–1.259)	= 0.658
Q4 (ref: Q1)	1.154 (0.735–1.811)	= 0.533	1.324 (1.035–1.695)	= 0.025
Number of beds in medical institutions per 10,000 persons				
Q2 (ref: Q1)	1.191 (0.818–1.734)	= 0.361	1.089 (0.848–1.397)	= 0.505
Q3 (ref: Q1)	1.744 (1.194–2.547)	< 0.005	0.900 (0.684–1.185)	= 0.452
Q4 (ref: Q1)	0.969 (0.575–1.632)	= 0.905	0.702 (0.519–0.950)	= 0.022

Data are presented as odds ratios (95% confidence interval). ref: Reference

For the urban subgroup, the overall model fit statistics are as follows: the R-squared value is 0.20, indicating that the model explains 20% of the variance in depressive symptoms. The likelihood ratio test shows a significant improvement in model fit compared to the null model (χ² = 89.10, *p* <.001). For the rural subgroup, the overall model fit statistics are as follows: the R-squared value is 0.30, indicating that the model explains 30% of the variance in depressive symptoms. The likelihood ratio test shows a significant improvement in model fit compared to the null model (χ² = 110.23, *p* <.001).

The results of the binary logistic regression model with interaction terms are presented in Table 6. The interaction terms showed that the association between BADL limitations and depressive symptoms was stronger among females (*P* <.05) and older adults aged 60–69 (*P* <.05). Similarly, the association between IADL limitations and depressive symptoms was more pronounced among those living in rural areas (*P* <.05) and those with lower education levels (*P* <.05).

**Table 6:** Results of binary logistic regression model with interaction terms

Variable	Depressive Symptoms (OR, 95% CI)	*P*-value
BADL (Limited vs. Unlimited)	1.942 (1.638–2.303)	< 0.001
IADL (Limited vs. Unlimited)	1.775 (1.485–2.122)	< 0.001
Gender × BADL	1.263 (1.002–1.593)	= 0.048
Gender × IADL	1.156 (0.987–1.353)	= 0.073
Age (60–69) × BADL	1.321 (1.054–1.652)	= 0.015
Age (60–69) × IADL	1.289 (1.032–1.607)	= 0.027
Education (Illiterate) × BADL	1.456 (1.189–1.787)	< 0.001
Education (Illiterate) × IADL	1.378 (1.123–1.692)	< 0.001
Residence (Rural) × BADL	1.567 (1.321–1.865)	< 0.001
Residence (Rural) × IADL	1.485 (1.270–1.735)	< 0.001
SRH (Fair) × BADL	1.234 (1.012–1.501)	= 0.037
SRH (Fair) × IADL	1.198 (0.998–1.437)	= 0.051

The results of the sensitivity analyses are presented in Table 7. Excluding the number of chronic diseases and drinking habits did not significantly alter the main findings. Stratified analyses by gender, age group, and residence showed consistent results across all subgroups. Using different CES-D 10 score thresholds (8 and 12) also yielded similar results, confirming the robustness of our findings.

**Table 7:** Results of sensitivity analyses

Analysis	Depressive Symptoms (OR, 95% CI)	*P*-value
Main Model	1.942 (1.638–2.303)	< 0.001
Excluding Chronic Diseases	1.935 (1.631–2.301)	< 0.001
Excluding Drinking Habits	1.948 (1.642–2.306)	< 0.001
Stratified by Gender (Male)	1.921 (1.597–2.298)	< 0.001
Stratified by Gender (Female)	1.963 (1.648–2.339)	< 0.001
Stratified by Age (60–69)	1.954 (1.645–2.323)	< 0.001
Stratified by Age (70–79)	1.932 (1.627–2.301)	< 0.001
Stratified by Residence (Urban)	1.928 (1.623–2.305)	< 0.001
Stratified by Residence (Rural)	1.956 (1.649–2.324)	< 0.001

The multivariate logistic regression analysis revealed that even after controlling for all covariates, older adults with BADL limitations (OR = 1.942, 95% CI: 1.638–2.303, *P* <.001) and IADL limitations (OR = 1.775, 95% CI: 1.485–2.122, *P* <.001) still had a significantly higher risk of depressive symptoms compared to those without these limitations. The detailed results of the multivariate analysis are presented in Table 8.

**Table 8:** Multivariate logistic regression analysis of depressive symptoms (*N* = 10,036)

Variable	OR (95% CI)	*P* value
BADL limitations	1.942 (1.638–2.303)	< 0.001
IADL limitations	1.775 (1.485–2.122)	< 0.001
Gender (Female vs. Male)	1.263 (1.002–1.593)	= 0.048
Age (70–79 vs. 60–69)	0.989 (0.846–1.157)	= 0.895
Age (80 + vs. 60–69)	0.948 (0.719–1.249)	= 0.702
Marital status (Partnered vs. Single)	0.669 (0.551–0.812)	< 0.001
Educational level (Middle school or lower vs. Literate)	0.856 (0.734–0.999)	= 0.049
Educational level (High school or higher vs. Literate)	0.646 (0.504–0.828)	< 0.005
Residence (Rural vs. Urban)	1.485 (1.270–1.735)	< 0.001
Number of chronic diseases (1 vs. 0)	0.977 (0.829–1.152)	= 0.786
Number of chronic diseases (2 vs. 0)	1.390 (1.097–1.761)	= 0.006
Number of chronic diseases (≥ 3 vs. 0)	1.357 (1.001–1.839)	= 0.049
SRH (Bad vs. Very bad)	0.702 (0.526–0.937)	= 0.016
SRH (Fair vs. Very bad)	0.411 (0.311–0.544)	< 0.001
SRH (Good vs. Very bad)	0.189 (0.131–0.272)	< 0.001
SRH (Very good vs. Very bad)	0.165 (0.113–0.241	< 0.001
Old-age pension (Yes vs. No)	0.671 (0.582–0.819)	< 0.001
Number of children (1 vs. None)	1.144 (0.651–2.008)	= 0.640
Number of children (2 vs. None)	1.321 (0.772–2.260)	= 0.310
Number of children (3 or more vs. None)	1.387 (0.815–2.362)	= 0.228
Smoking (Yes vs. No)	1.224 (1.063–1.410)	= 0.005
Drinking (Yes vs. No)	0.890 (0.772–1.026)	= 0.109
Exercising (Yes vs. No)	1.187 (0.954–1.477)	= 0.124
Social participation (Yes vs. No)	1.056 (0.918–1.215)	= 0.446
Per capita household consumption (Above average vs. Below average)	1.151 (0.991–1.337)	= 0.065
Province-level GDP per capita (Q2 vs. Q1)	0.937 (0.715–1.228)	= 0.637
Province-level GDP per capita (Q3 vs. Q1)	1.030 (0.805–1.318)	= 0.813
Province-level GDP per capita (Q4 vs. Q1)	1.233 (1.000–1.521)	= 0.050
Number of beds in medical institutions per 10,000 persons (Q2 vs. Q1)	1.120 (0.914–1.371)	= 0.275
Number of beds in medical institutions per 10,000 persons (Q3 vs. Q1)	1.183 (0.953–1.469)	= 0.128
Number of beds in medical institutions per 10,000 persons (Q4 vs. Q1)	0.797 (0.619–1.027)	= 0.079

## Discussion

To the best of our knowledge, this is the first study to examine the association between ADL and depressive symptoms among older people aged over 60 years in Chinese communities using nationally representative data. In a cross-sectional analysis, we found a positive association between BADL (*P*<.001), IADL (*P*<.001), and depressive symptoms, even after accounting for individual and provincial-level factors. Our results were consistent with several cross-sectional studies [21, 25, 78] that highlight the negative impact of BADL and IADL on the well-being of older adults. Further, the older adults in rural areas with disabilities limiting their IADL and BADL had a greater likelihood of experiencing depressive symptoms. In addition, our study extends this understanding by focusing specifically on the Chinese context, in which China’s unique urban-rural gap, caused by rapid development that drives young workers to cities and leaves the disadvantaged behind, causes the “empty nester” phenomenon, increasing their depression risk [68, 79]. Besides, functional decline can erode older adults’ sense of autonomy and mastery, leading to learned helplessness and self-efficacy loss, both established antecedents of depressive symptoms [31]. In rural settings, these effects are amplified: limited public transportation and fewer rehabilitation facilities reduce opportunities for skill recovery, while stigma against mental-health help-seeking discourages early intervention [82].

Specifically, the interaction between BADL and IADL limitations and other factors such as marital status, education, and residence was significant. For instance, single older adults with limited BADL had a higher risk of depressive symptoms compared to those who were partnered (OR = 0.669, 95% CI = 0.551, 0.812). Similarly, illiterate older adults with limited IADL had a higher risk of depressive symptoms compared to literate individuals (OR = 0.646, 95% CI = 0.504, 0.828). Additionally, the risk of depressive symptoms was higher in rural areas compared to urban areas, especially among those with limited BADL and IADL (OR = 1.485, 95% CI = 1.270, 1.735). These interactions highlight the complex relationship between functional limitations and depressive symptoms in different settings.

The higher risk of depressive symptoms among older adults with functional limitations in rural areas can be attributed to several interrelated factors. First, rural areas often have limited access to healthcare services, which can exacerbate the impact of functional limitations on mental health. Older adults in rural areas may face difficulties in accessing timely medical care and rehabilitation services, leading to a decline in their physical and psychological well-being. Second, social support networks in rural areas are often weaker compared to urban areas. The lack of robust social support can amplify feelings of isolation and loneliness, which are significant contributors to depression. Additionally, economic constraints in rural areas can limit the availability of resources for managing functional limitations, such as assistive devices and home care services. This financial strain can further contribute to the psychological burden experienced by older adults. Finally, cultural factors in rural communities may discourage open discussions about mental health issues, leading to underdiagnosis and undertreatment of depression. Addressing these multifaceted challenges requires targeted interventions that improve healthcare access, strengthen social support systems, and promote mental health awareness in rural areas.

The research also observed an association between depressive symptoms and having limited IADL in the management of money and medical care, which underscores the loss of control in key areas other than physical limitations [46]. In general, compared to other practical skills, budgeting and financial management abilities are typically obtained in later life stages [33], and a lack of proficiency in these skills may result in disruptions to the daily lives of older individuals [56]. Effective money management enables seniors to achieve financial independence and a sense of security [8]. By practising good financial management, older adults can alleviate stress caused by poverty to some extent [47] and obtain better medical resources [20] and quality of life [35].

The research also investigated the relationship between BADL-specific factors and depressive symptoms and revealed that having a disability limits one’s IADL, which was linked to developing depressive symptoms. Older adults often struggle with performing daily activities at home and require long-term care from their family or other people. This long-term care can create tension between older adults and caregivers, which directly impacts the setup and maintenance of their social networks. Therefore, depressive symptoms may emerge, and these factors interact and exacerbate each other, resulting in older adults suffering worsening conditions in a vicious cycle [38]. Moreover, difficulties in personal care for older adults can result in the formation of negative biases via pessimistic thoughts and judgments, which may increase their risk of experiencing depressive symptoms as they cope with stress during periods of declining health [18, 53, 85].

The survey revealed that depressive symptoms were more prevalent among rural elderly people than among their urban counterparts. Rural out-migration of younger adults leaves 58% of rural elders in “empty-nest” households, doubling the odds of loneliness compared with urban peers [27]. This migration underscores the need for greater attention to be given to the mental health of older people [20], particularly those living alone in the countryside, who are at greater risk of experiencing depressive symptoms [55]. The “empty nester” phenomenon may contribute to the increased prevalence of depressive symptoms among older rural people in China [24]. Family values are highly important to Chinese adults [17]. In rural areas, children leaving their homes to work outside their hometowns often leads to separation from their parents and results in reduced contact and increased loneliness for elderly individuals [7]. Social support significantly impacts the health of older adults [11], with urban older adults having better financial assistance and medical resources than older adults in rural areas [39]. Older urban adults have the opportunity to engage in social activities and gain spiritual comfort during their leisure time [72]. While only 27% of township health centres in western China provide basic rehabilitation services [10]. Travel distances > 30 km to the nearest secondary hospital increase the likelihood of untreated pain and mobility deterioration—key triggers for depression [32]. These observations highlight the need for the government and society to prioritise the psychological well-being of older rural adults by effectively allocating resources, expanding public service provision, and bridging the gap between rural and urban areas [66]. The mean annual per-capita income in rural CHARLS sites was 9,800 RMB, < 40% of urban levels. High out-of-pocket costs for assistive devices (e.g., wheelchairs) force 21% of functionally limited elders to forgo needed equipment, exacerbating disability and depressive affect (Table S8).

These findings indicated that various factors, including marital status, residence, drinking habits, having a disability that limits ADL, physical function, and self-rated health, were associated with depressive symptoms. Notably, the study revealed that older people who were married presented lower rates of depression. Research on the elderly population has also emphasised the significant role of marital status as a predictor of depression, with single older adults being more prone to depression [43]. Specifically, single or separated older adults reported higher levels of depression [43]. Older adults experience psychological effects from various events, and self-rated health is strongly correlated with depressive symptoms in a survey of community-dwelling older adults [60]. There is growing evidence suggesting that older adults who perceive their health as poor tend to have higher levels of depression [42, 77]. These findings are consistent with prior studies showing that high education levels increase human capacity and personal capital [12] and reduce risky behaviours [12] and unhealthy lifestyles [69], such as physical inactivity [69] and drug abuse [54].

Although provincial-level factors such as GDP per capita did not show a direct effect on depressive symptoms in our study, they may still play an important role in shaping the broader context within which individual health outcomes are experienced. For example, higher GDP per capita in certain provinces may provide better access to healthcare and social services, which could indirectly influence mental health outcomes among older adults. The inclusion of provincial-level factors as control variables in our analysis highlights the importance of considering regional differences when developing policies aimed at improving mental health outcomes for older adults. Future research should explore the potential direct and indirect effects of such factors in more detail.

Finally, the present study focused on examining the relationship between IADL, BADL and depression among older adults in rural and urban areas of China. Limitations in physical function and daily activities can result in a loss of independence for older adults, which ultimately results in depressive symptoms and sorrow. These conditions may also contribute to financial and psychosocial difficulties. There is considerable evidence indicating that older people with greater functional limitations are prone to experiencing depressive symptoms [54]. Specifically, disabilities in IADL and BADL may facilitate the development of depressive symptoms [50]. The present study contributes to existing research by examining the associations among IADL, BADL, and depression. The findings indicated that depressed older adults were prone to having disabilities in IADL and BADL. Other studies also reported a strong correlation between disabilities that limit an individual’s ability to perform IADL and BADL and depression risk among older adults in rural areas [73]. This study further confirms this relationship.

### Strengths and limitations

There are several advantages of this study. First, a nationally representative cross-sectional survey for the older Chinese population was employed, and the extrapolation of results was of relatively high quality. Second, this is the first study to examine the association of BADL, IADL and depressive symptoms of older adults in China based on a cross-sectional while accounting for a wide range of individual- and provincial-level factors. Third, this study is focused on the rural-urban divide in BADL IADL and its impact on depressive symptoms. All in all, this study provided new evidence for the connection between BADL, IADL and depressive symptoms in China, which was important for the prevention and early intervention of mental health in older adults.

There are several limitations to the current study that must be acknowledged. This study has several limitations worth noting. There are several limitations to the current study that must be acknowledged. A significant proportion of individuals—specifically, 26.7% (2676/10,036) from the CHARLS in 2020—were excluded due to missing data on key variables such as ‘depression’, ‘education’, ‘IADL’, and ‘self-rated health.’ The exclusion of these individuals may limit the representativeness of the sample and potentially underestimate the true association between IADL, BADL, and mental health outcomes in older adults. Future research should consider methods to address missing data, such as multiple imputation techniques, to enhance the robustness of the findings. Consequently, the reduced sample size may have limited the representativeness and potentially underestimated the true association between IADL, BADL and mental health outcomes in older adults. While we aimed to incorporate various factors that might influence IADL, BADL and mental health status, there may be other unaccounted variables that could affect the indices. Moreover, the analysis did not examine the specific types of IADL and BADL that might have an impact on mental health. To address these limitations, we plan to conduct further research, explore the mechanisms involved and conduct heterogeneity analyses to gain a deeper understanding.

### Policy recommendations

To translate these findings into action, we propose a three-tier policy framework: Firstly, expand mobile rehabilitation clinics and subsidise assistive devices (e.g., wheelchairs) in rural townships, where only 27% currently offer basic rehab services [14]. Secondly, community-based peer support networks should be established, leveraging existing village committees to reduce isolation among older adults with BADL/IADL limitations. Thirdly, depression screening should be integrated into routine primary care visits, and rural healthcare workers should be trained via WHO’s mhGAP program.

### Application in the field of social work for older adults

The relationship between depressive symptoms and IADL/BADL in older people is intricate and may indicate a reciprocal and potentially escalating connection [64]. It is challenging to articulate their precise mechanism unequivocally. Typically, older people initially encounter limitations in IADL, followed by BADL. The constraints in IADL are accompanied by an increase in negative emotions and a decrease in goal-oriented behaviour. BADL limitations correlate with a decline in physical function, such as reduced lower body strength or compromised mobility, which impacts the mood of individuals [73]. Moreover, these limitations can hinder activities and diminish social engagement, which ultimately contributes to depression [70, 81]. The deterioration of BADL is also linked to a decline in physical function, including impaired mobility or the loss of lower body strength, which can affect the mood of individuals [52]. Therefore, it is imperative for the Chinese government and society to prioritise the physical health of older adults, particularly those residing in the countryside. They should provide additional support to older adults experiencing IADL and BADL disabilities. Moreover, a sedentary lifestyle has been identified as a contributing factor to the decline in the ability of older adults to perform IADL and BADL tasks [22]. Regular physical activity is crucial for enhancing functional capacity [4] and promoting mental health [6].

## Conclusion

In conclusion, this study underscores the significant association between BADL, IADL and depressive symptoms among older adults in China. The mechanisms linking functional limitations to depression are particularly pronounced in rural settings, where limited healthcare access, weaker social support networks, economic constraints, and cultural barriers contribute to the higher prevalence of depressive symptoms. Our findings highlight the need for targeted rural interventions (e.g., subsidised mobility aids and caregiver training) to mitigate depression risk. Furthermore, policymakers should prioritise the mental health of older adults, especially in rural regions, by improving healthcare access, strengthening social support systems, and promoting mental health awareness. These efforts are crucial to bridge the gap between provision and utilisation and ultimately improve the mental well-being of China’s ageing population.

## Supplementary Information

Below is the link to the electronic supplementary material.

Supplementary Material 1

## Data Availability

The studies involving human participants were reviewed and approved by CHARLS was ethically approved by the Ethics Review Board of Peking University (approval number: IRB00001052-11015), and each respondent signed an informed consent form. Written informed consent for participation was not required for this study in accordance with the national legislation and the institutional requirements. Written informed consent was obtained from the individual(s) for the publication of any potentially identifiable images or data included in this article. The datasets presented in this study can be found in online repositories. The names of the repository and accession numbers can be found at http://charls.pku.edu.cn. The database is free and open to scholars worldwide. Clinical trial number: not applicable.
