# Supplementary material for: Association between activities of daily living and depression symptoms among older adults in China: A nationally representative cross-sectional survey
Source: BMC Psychol. 2025 Aug 30;13:989. doi: 10.1186/s40359-025-03223-9 (PMC12398042; doi:10.1186/s40359-025-03223-9)
Supplement: Supplementary file 1 — Supplementary Material 1 [file 40359_2025_3223_MOESM1_ESM.docx]

**Association between activities of daily living and depression symptoms among older adults in China: A nationally representative cross-sectional survey**

**Supplementary material**

Catalog

[Figure S1 Flow chart of study participants 2](#_Toc132922157)

[Table S1 STROBE Statement—checklist of items that should be included in reports of observational studies. 3](#_Toc132922159)

[Table S2 The item of the Center for Epidemiological Survey Depression Scale 6](#_Toc132922160)

[Table S3 Univariate analysis. 8](#_Toc132922162)

# Figure S1 Flow chart of study participants


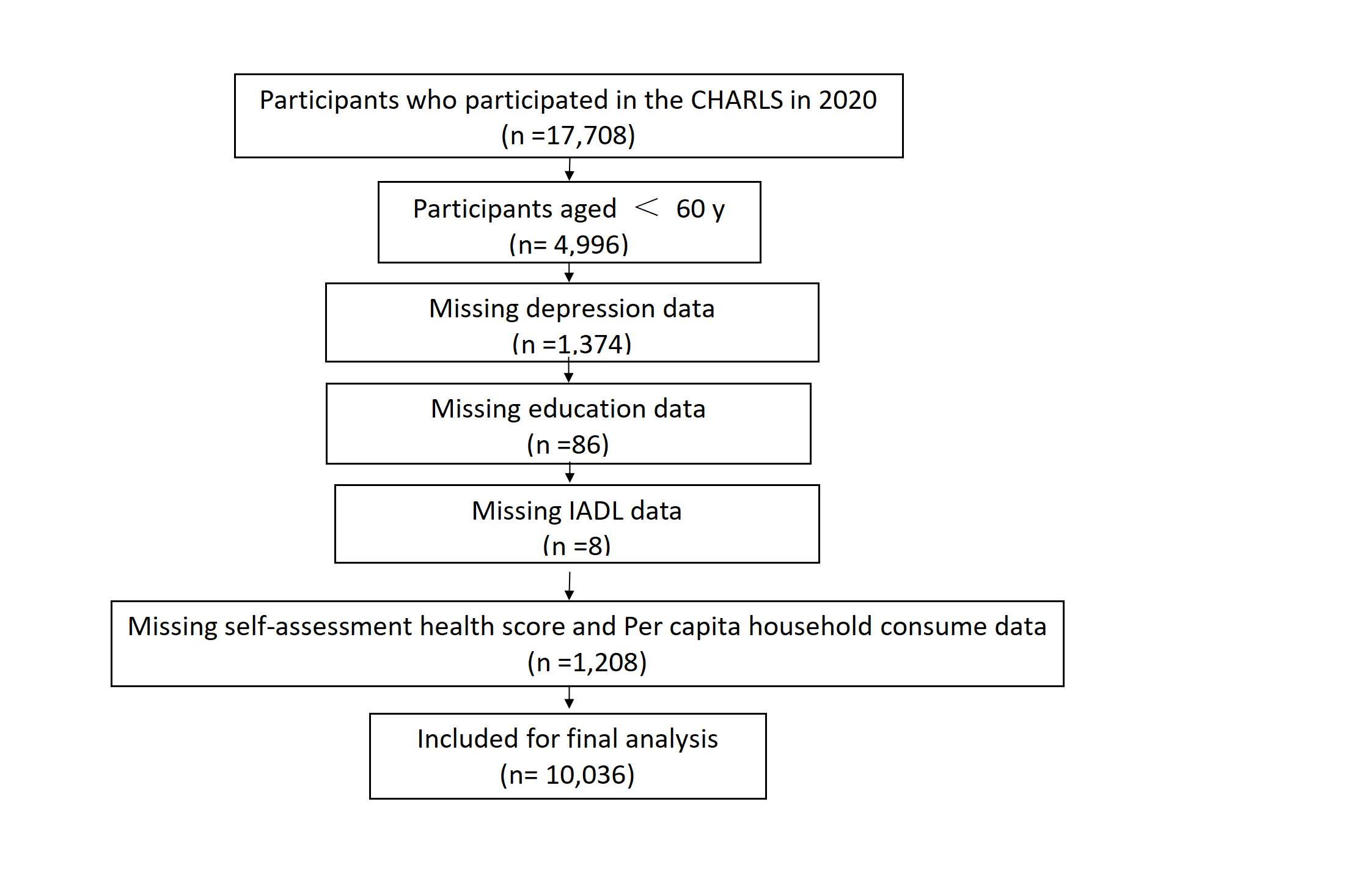


# Table S1 STROBE Statement—checklist of items that should be included in reports of observational studies.

|  | Item No | Recommendation |  |
| --- | --- | --- | --- |
| **Title and abstract** | 1 | (*a*) Indicate the study’s design with a commonly used term in the title or the abstract | page 1 |
|  |  | (*b*) Provide in the abstract an informative and balanced summary of what was done and what was found | Abstract (page 1) |
| Introduction | | |  |
| Background/rationale | 2 | Explain the scientific background and rationale for the investigation being reported | Page 2-3 |
| Objectives | 3 | State specific objectives, including any prespecified hypotheses | Page 3 |
| Methods | | |  |
| Study design | 4 | Present key elements of study design early in the paper | Page 4 |
| Setting | 5 | Describe the setting, locations, and relevant dates, including periods of recruitment, exposure, follow-up, and data collection | Page 4 |
| Participants | 6 | (*a*) *Cross-sectional study*—Give the eligibility criteria, and the sources and methods of selection of participants | Page 4 |
|  |  | (*b*) *Cohort study*—For matched studies, give matching criteria and number of exposed and unexposed  *Case-control study*—For matched studies, give matching criteria and the number of controls per case | Not applicable*.* |
| Variables | 7 | Clearly define all outcomes, exposures, predictors, potential confounders, and effect modifiers. Give diagnostic criteria, if applicable | Page 4-5 |
| Data sources/ measurement | 8* | For each variable of interest, give sources of data and details of methods of assessment (measurement). Describe comparability of assessment methods if there is more than one group | Page 4-5 |
| Bias | 9 | Describe any efforts to address potential sources of bias | None |
| Study size | 10 | Explain how the study size was arrived at | Not applicable. |
| Quantitative variables | 11 | Explain how quantitative variables were handled in the analyses. If applicable, describe which groupings were chosen and why | Statistical analysis  (Page 6-7) |
| Statistical methods | 12 | (*a*) Describe all statistical methods, including those used to control for confounding | Statistical analysis  (Page 5-6) |
|  |  | (*b*) Describe any methods used to examine subgroups and interactions | Statistical analysis  (Page 5-6) |
|  |  | (*c*) Explain how missing data were addressed | Page 4 |
|  |  | (*d*) *Cross-sectional study*—If applicable, describe analytical methods taking account of sampling strategy | Page 4 |
|  |  | (*e*) Describe any sensitivity analyses | None |

Continued on next page

| Results | | |  |
| --- | --- | --- | --- |
| Participants | 13* | (a) Report numbers of individuals at each stage of study—eg numbers potentially eligible, examined for eligibility, confirmed eligible, included in the study, completing follow-up, and analysed | Page 5 |
|  |  | (b) Give reasons for non-participation at each stage | Figure S1 |
|  |  | (c) Consider use of a flow diagram | Figure S1 |
| Descriptive data | 14* | (a) Give characteristics of study participants (eg demographic, clinical, social) and information on exposures and potential confounders | Table S3 |
|  |  | (b) Indicate number of participants with missing data for each variable of interest | Table 1 |
|  |  | (c) *Cohort study*—Summarise follow-up time (eg, average and total amount) | Not applicable |
| Outcome data | 15* | *Cohort study*—Report numbers of outcome events or summary measures over time | Not applicable |
|  |  | *Case-control study—*Report numbers in each exposure category, or summary measures of exposure | Not applicable |
|  |  | *Cross-sectional study—*Report numbers of outcome events or summary measures | Page 6-7 |
| Main results | 16 | (*a*) Give unadjusted estimates and, if applicable, confounder-adjusted estimates and their precision (eg, 95% confidence interval). Make clear which confounders were adjusted for and why they were included | Page 6-7 |
|  |  | (*b*) Report category boundaries when continuous variables were categorized | Table S4 |
|  |  | (*c*) If relevant, consider translating estimates of relative risk into absolute risk for a meaningful time period | None |
| Other analyses | 17 | Report other analyses done—eg analyses of subgroups and interactions, and sensitivity analyses | Table S6 |
| Discussion | | |  |
| Key results | 18 | Summarise key results with reference to study objectives | Page 7 |
| Limitations | 19 | Discuss limitations of the study, taking into account sources of potential bias or imprecision. Discuss both direction and magnitude of any potential bias | Page 10 |
| Interpretation | 20 | Give a cautious overall interpretation of results considering objectives, limitations, multiplicity of analyses, results from similar studies, and other relevant evidence | Page 7-10 |
| Generalisability | 21 | Discuss the generalisability (external validity) of the study results | Page 10 |
| Other information | | |  |
| Funding | 22 | Give the source of funding and the role of the funders for the present study and, if applicable, for the original study on which the present article is based | Page 11 |

*Give information separately for cases and controls in case-control studies and, if applicable, for exposed and unexposed groups in cohort and cross-sectional studies.

**Note:** An Explanation and Elaboration article discusses each checklist item and gives methodological background and published examples of transparent reporting. The STROBE checklist is best used in conjunction with this article (freely available on the Web sites of PLoS Medicine at http://www.plosmedicine.org/, Annals of Internal Medicine at http://www.annals.org/, and Epidemiology at http://www.epidem.com/). Information on the STROBE Initiative is available at www.strobe-statement.org.

# Table S2 The item of the Center for Epidemiological Survey Depression Scale

| 1. I was bothered by things that don’t usually bother me. | 1. Rarely or none of the time (*<* 1 day) |
| --- | --- |
|  | 2. Some or a little of the time (1 *−* 2 days) |
|  | 3. Occasionally or a moderate amount of the time (3 *−* 4 days) |
|  | 4. Most or all of the time (5 *−* 7 days) |
| 1. I had trouble keeping my mind on what I was doing. | 1. Rarely or none of the time (*<* 1 day) |
|  | 2. Some or a little of the time (1 *−* 2 days) |
|  | 3. Occasionally or a moderate amount of the time (3 *−* 4 days) |
|  | 4. Most or all of the time (5 *−* 7 days) |
| 1. I felt depressed. | 1. Rarely or none of the time (*<* 1 day) |
|  | 2. Some or a little of the time (1 *−* 2 days) |
|  | 3. Occasionally or a moderate amount of the time (3 *−* 4 days) |
|  | 4. Most or all of the time (5 *−* 7 days) |
| 1. I felt everything I did was an effort. | 1. Rarely or none of the time (*<* 1 day) |
|  | 2. Some or a little of the time (1 *−* 2 days) |
|  | 3. Occasionally or a moderate amount of the time (3 *−* 4 days) |
|  | 4. Most or all of the time (5 *−* 7 days) |
| 1. I felt hopeful about the future. ^†^ | 1. Rarely or none of the time (*<* 1 day) |
|  | 2. Some or a little of the time (1 *−* 2 days) |
|  | 3. Occasionally or a moderate amount of the time (3 *−* 4 days) |
|  | 4. Most or all of the time (5 *−* 7 days) |
| 1. I felt fearful. | 1. Rarely or none of the time (*<* 1 day) |
|  | 2. Some or a little of the time (1 *−* 2 days) |
|  | 3. Occasionally or a moderate amount of the time (3 *−* 4 days) |
|  | 4. Most or all of the time (5 *−* 7 days) |
| 1. My sleep was restless. | 1. Rarely or none of the time (*<* 1 day) |
|  | 2. Some or a little of the time (1 *−* 2 days) |
|  | 3. Occasionally or a moderate amount of the time (3 *−* 4 days) |
|  | 4. Most or all of the time (5 *−* 7 days) |
| 1. I was happy. ^†^ | 1. Rarely or none of the time (*<* 1 day) |
|  | 2. Some or a little of the time (1 *−* 2 days) |
|  | 3. Occasionally or a moderate amount of the time (3 *−* 4 days) |
|  | 4. Most or all of the time (5 *−* 7 days) |
| 1. I felt lonely. | 1. Rarely or none of the time (*<* 1 day) |
|  | 2. Some or a little of the time (1 *−* 2 days) |
|  | 3. Occasionally or a moderate amount of the time (3 *−* 4 days) |
|  | 4. Most or all of the time (5 *−* 7 days) |
| 1. I could not get “going” | 1. Rarely or none of the time (*<* 1 day) |
|  | 2. Some or a little of the time (1 *−* 2 days) |
|  | 3. Occasionally or a moderate amount of the time (3 *−* 4 days) |
|  | 4. Most or all of the time (5 *−* 7 days) |

^†^ Entries 5 and 8 are reverse scoring.

# Table S3 Univariate analysis

| Province level |  |  |  |
| --- | --- | --- | --- |
|  | N | Mean | P value |
| GDP per ca-pita |  |  | P＜.001 |
| The east | 2715 | 87752.52 |  |
| The middle | 2585 | 59787.21 |  |
| The Northeast | 526 | 52435.20 |  |
| The west | 2866 | 56316.88 |  |
| Total | 8692 | 66933.17 |  |
| Number of beds in medical institutions per 10,000 persons |  |  | P＜.001 |
| The east | 2715 | 57.481 |  |
| The middle | 2585 | 68.251 |  |
| The Northeast | 526 | 74.801 |  |
| The west | 2866 | 70.608 |  |
| Total | 8692 | 66.060 |  |

Data are presented as odds ratio (95% confidence interval).
